# Human Genetics and the Causal Role of Lipoprotein(a) for Various Diseases

**DOI:** 10.1007/s10557-016-6648-3

**Published:** 2016-02-20

**Authors:** Florian Kronenberg

**Affiliations:** Division of Genetic Epidemiology, Department of Medical Genetics, Molecular and Clinical Pharmacology, Medical University of Innsbruck, Schöpfstr. 41, 6020 Innsbruck, Austria

**Keywords:** lipoprotein(a), Apolipoprotein(a), Cardiovascular disease, Copy number variation, Association study, Mendelian randomization

## Abstract

Lipoprotein(a) [Lp(a)] is a highly atherogenic lipoprotein that is under strong genetic control by the *LPA* gene locus. Genetic variants including a highly polymorphic copy number variation of the so called kringle IV repeats at this locus have a pronounced influence on Lp(a) concentrations. High concentrations of Lp(a) as well as genetic variants which are associated with high Lp(a) concentrations are both associated with cardiovascular disease which very strongly supports causality between Lp(a) concetrations and cardiovascular disease. This method of using a genetic variant that has a pronounced influence on a biomarker to support causality with an outcome is called Mendelian randomization approach and was applied for the first time two decades ago with data from Lp(a) and cardiovascular disease. This approach was also used to demonstrate a causal association between high Lp(a) concentrations and aortic valve stenosis, between low concentrations and type-2 diabetes mellitus and to exclude a causal association between Lp(a) concentrations and venous thrombosis. Considering the high frequency of these genetic variants in the population makes Lp(a) the strongest genetic risk factor for cardiovascular disease identified so far. Promising drugs that lower Lp(a) are on the horizon but their efficacy in terms of reducing clinical outcomes still has to be shown.

## Introduction

Lipoprotein(a) [Lp(a)] consists of an LDL particle and the glycoprotein apolipoprotein(a) [apo(a)] which is linked to the apolipoprotein B from LDL by a single disulfide bond [1]. It is synthesized in the liver but the site and mechanism of catabolism is discussed controversially: no receptor specific for Lp(a)/apo(a) has been described but several observations point to a role of the kidney in Lp(a) catabolism [1–3].

**Fig. 1:**
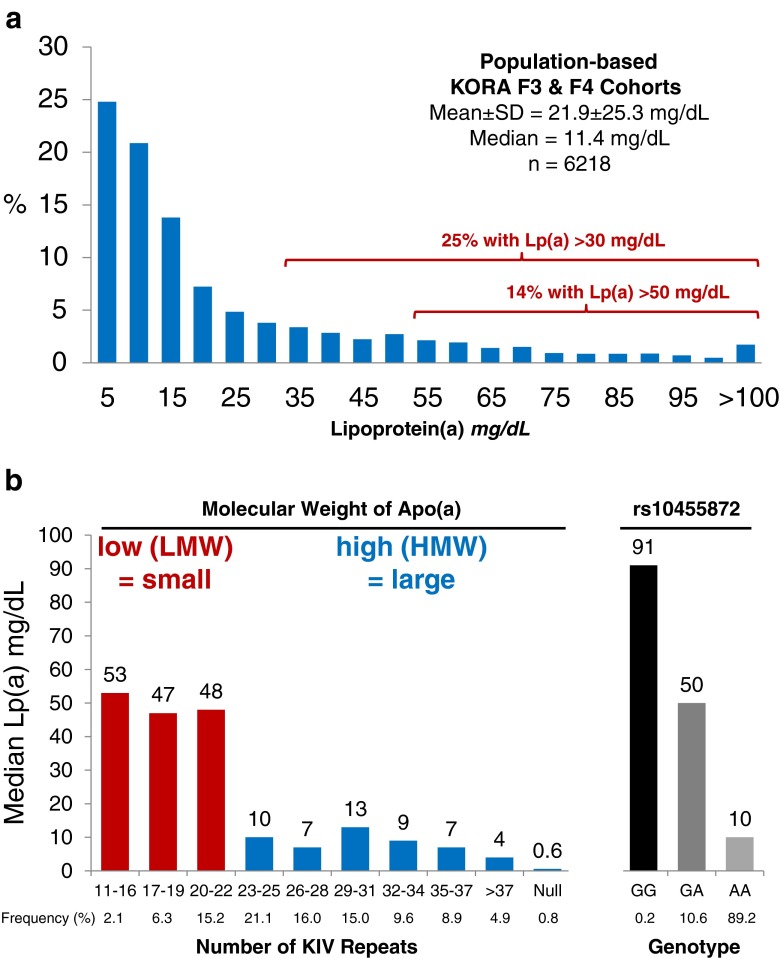
Panel a, distribution of Lp(a) concentration in 6218 individuals from the two population-based studies KORA F3 and F4. Panel b, median Lp(a) concentrations in various groups of subjects stratified by the number of KIV repeats and genotypes of SNP rs10455872; 11–22 KIV repeats are considered as low molecular weight (LMW) or small apo(a) isoforms and those with >22 KIV repeats are considered as high molecular weight (HMW) or large apo(a) isoforms. Figure adapted and reprinted with permission of reference [107]

An astonishing characteristic of Lp(a) is the more than 1000-fold range of concentrations between individuals from less than 0.1 mg/dL to more than 300 mg/dL with a skewed distribution in most populations (Fig. 1a). Lp(a) concentrations are not very much influenced by age, sex, fasting state, inflammation [4, 5] and lifestyle factors such as diet or physical activity but are under strict genetic control by the *LPA* gene locus and here especially by a size polymorphism of apo(a) caused by a variable number of kringle IV (KIV) repeats in the *LPA* gene [1, 6].

The physiological function of Lp(a) is still unclear. Medical interest in Lp(a) started when it was discovered that high Lp(a) plasma concentrations are associated with cardiovascular disease (CVD). The high homology of apo(a) and plasminogen [7] directed research to the fibrinolytic system and it was suggested that Lp(a) may act as a modulator of the balance between blood clotting and fibrinolysis. Numerous studies mostly done in vitro found that Lp(a) indeed interferes with the blood clotting/fibrinolytic cascades by e.g., inhibition of streptokinase and urokinase-mediated activation of plasminogen by the tissue-type plasminogen activator (t-PA), inhibition of t-PA in solution, fibrin and fibrinogen binding, competition with plasminogen and t-PA binding for soluble fibrinogen, competition with plasminogen for binding to cellular receptors, and enhancement of the plasminogen-activator-inhibitor PAI-1 activity (reviewed in [8]). From the more than 1000-fold interindividual range in Lp(a) concentrations one would expect major influences on the involved systems also in vivo but this has not been described convincingly.

An unexpected and intriguing observation is the binding of oxidized phospholipids (OxPl) to apo(a) of the Lp(a) particle [9, 10]. Levels of Lp(a) and OxPl in human plasma are highly correlated, suggesting that individuals with high Lp(a) have a higher binding capacity for OxPl and have more OxPl in their plasma. Lp(a) has therefore been proposed to function as a “sink” for OxPl [11]. Not unexpectedly this association also results in an association of OxPl levels with CVD [12, 13].

## *LPA* Gene and Structure of Lp(a)

To understand the genetics of Lp(a) one first has to understand the structure of the *LPA* gene and how this structure has developed during evolution. The *LPA* gene evolved by duplication and remodeling from the plasminogen (*PLG*) gene during primate evolution and is only present in old world monkeys and primates including humans [7, 14]. This limits research considerably since most animal models might be restricted due to the fact that these model organisms might miss besides the *LPA* gene also the other genes for the machinery involved in the synthesis and catabolism of Lp(a).

*PLG* contains five types of kringle domains called KI to KV and a protease domain. The human *LPA* gene does not have KI to KIII, but KIV, KV and the protease domain are present. The peculiarity for *LPA* is the KIV which has expanded and diversified by mutation into ten different types (KIV type 1–10). Within these ten different types the KIV-2 exists in multiple copies ranging from two to more than 40 repeats. Each of these repeats has a size of 5.6 kB which results in a highly polymorphic and informative copy number variation (CNV) with a heterozygosity of more than 95 % in most populations.

The KIV-2 CNV is transcribed into mRNA and translated into the apo(a) isoform protein. During the assembly to the Lp(a) particle, the apo(a) isoform binds covalently to apolipoprotein B of an LDL particle in a stoichiometric manner and forms the Lp(a) particle [15].

The *LPA* gene is highly expressed in the liver but not in other organs [7]. The regulation of expression is not very well understood. Transcription factor binding sites in the 5′-region of the *LPA* gene are known for HNF1α, HNF4α, sex hormones and acute phase inducers. A retinoid response element is present in the *LPA* promoter and an enhancer residing in a LINE element has been found in the intragenic region between *LPA* and *PLG* [16]. An Ets binding element in the human *LPA* promoter functions as an ELK-1 binding site that mediates repression of *LPA* transcription by FGF19 [17]. Interestingly, *LPA* gene expression is downregulated by bile acids and the effect is mediated by the farnesoid-X receptor (FXR), which represses hepatic *LPA* gene expression in humans by inferring with HNF4α. It was demonstrated that the *LPA* promoter contains a direct repeat element-1 (DR-1) between nucleotides −826 and −814 to which HNF4α binds promoting *LPA* transcription [18]. FXR competes with HNF4α for binding to the DR-1 element. Modulation of FXR has therefore been proposed as a potential target for Lp(a) lowering drugs.

## Genetic Variability and Influence on Lp(a) Concentrations

**Fig. 2:**
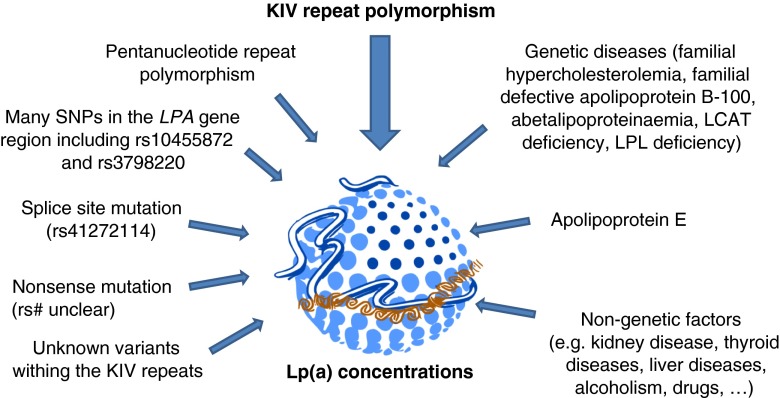
Overview on the most important genetic and non-genetic factors that have an influence on Lp(a) concentrations. The graphical illustration of the Lp(a) particle has been taken with permission from Hansi Weissensteiner (Medical University of Innsbruck)

Figure 2 provides an overview on the most important genetic and non-genetic factors that have an influence on Lp(a) concentrations. Some of them play different roles in various populations.

### KIV Repeat Polymorphism

The interindividual range of Lp(a) concentrations is very wide from less than 0.1 mg/dL to more than 300 mg/dL. Few people lack Lp(a) in their plasma [19, 20]. The broad range in the Lp(a) distribution is known for all populations and is highly skewed towards low levels in most ethnic groups. Figure 1a demonstrates in a population-based study from Southern Germany that roughly 50 % of the individuals have concentrations below 10 mg/dL. Approximately 25 % of this typical population have concentrations above 30 mg/dL and 14 % have concentrations above 50 mg/dL. Both thresholds have been considered to be associated with an increased risk for cardiovascular disease [1, 21]. In contrast to European and Asian populations sub-Saharian Africans show markedly higher Lp(a) concentrations with a distribution that is closer to a Gaussian distribution.

These substantial differences between individuals are to a large extent genetically determined. Early family studies established the genetic nature of the trait and twin studies found that the heritability of Lp(a) is very high, exceeding 90 % in populations of European and African descent [22, 23]. Lp(a) is therefore the lipoprotein with the strongest genetic control. The discovery of the size polymorphism of apo(a) in serum [24] and KIV-2 CNV in the *LPA* gene [6, 25–27] resulted in the identification of the *LPA* gene as the major gene for Lp(a) levels. Association and sib-pair linkage studies [27–31] revealed very soon that the KIV repeat size polymorphism explains a major part of this variance ranging from 30 to 70 % depending on the ethnic population [1]. Individuals expressing a low number of K-IV repeats resulting in so-called small apo(a) isoforms (up to 22 KIV repeats) have on average markedly higher Lp(a) concentrations than individuals carrying only large apo(a) isoforms (more than 22 KIV repeats) (Fig. 1b).

### Other Genetic Variants Besides the KIV Repeat Polymorphism

Despite this pronounced inverse correlation between the number of KIV repeats and the Lp(a) concentrations, there is still a wide variability in Lp(a) concentrations within each KIV repeat group. That means isoforms of the same size differ widely in concentrations. This suggests that besides non-genetic factors other genetic variants than the KIV repeats are major contributors to Lp(a) concentration variation and that there are pronounced differences between ethnicities. And indeed several dozens of genetic variants within the *LPA* gene region have been described in the meanwhile and for most of them, the functional consequences are not yet known. This has to be seen in the context that a genetic variant can be in strong linkage disequilibrium with the real causal variant and without functional studies it will be hard to determine which variant is functionally responsible for Lp(a) concentration variability. One of the first additional polymorphisms besides the KIV repeat polymorphism described to be associated with Lp(a) concentrations was a pentanucleotide repeat polymorphism in the promoter region of the *LPA* gene [32, 33]. This polymorphism explains up to 14 % of the Lp(a) variation in Europeans but shows no association in Black Africans [32]. However, functional promoter studies suggested that causal variants in linkage disequilibrium with the pentanucleotide repeat polymorphism rather than pentanucleotide repeat polymorphism itself are contributing to Lp(a) concentrations [34, 35].

There are two SNPs known which are functionally characterized and which result in non-expressed apo(a) alleles. A +1 donor splice site mutation (rs41272114) with a frequency of about 6 % in Europeans results in alternate splicing leading to a truncated apo(a) isoform with congenital deficiency of Lp(a) [19, 36]. Another SNP in the first exon of KIV type-2 introduces a stop codon resulting in a truncated apo(a) protein. This nonsense mutation causing a null allele is observed in a frequency of 2 % in Europeans [20].

Besides these well characterized variants several dozens of SNPs in the wider *LPA* region have recently been brought to the attention of the scientific community [37]. Many of them show pronounced associations with Lp(a) concentrations (e.g., rs10455872 and rs3798220) but the functional significance is not yet clear. Furthermore, since sequencing of the highly repetitive and large KIV type-2 repeat is until now hard to accomplish, we might expect some surprises on genetic variants in those regions which could have a pronounced effect on Lp(a) concentrations.

### Genome-Wide Association Studies (GWAS)

The search for Lp(a)-modifying genes using genome-wide association studies (GWAS) is in progress. GWAS performed up to now were strongly limited by sample size, focus on certain subgroups (e.g., diabetes mellitus, population isolates) or by the use of a specialized candidate gene-chip. Due to these limitations they were only able to identify the well-known region on chromosome 6q27 harboring *LPA*, *PLG*, *SLC22A3* [38–42] and very recently the *APOE* gene locus in African Americans [43]. This finding will require confirmation in different populations and functional elucidation. It will require a large number of samples to identify further genes contributing to Lp(a) levels, should such loci exist. A conditional analysis adjusted for the effects of the *LPA* locus and especially the apo(a) isoform size will tremendously increase the power of such GWAS.

### Familial Hypercholesterolemia

Two- to three-fold elevated Lp(a) levels were observed in patients with familial hypercholesterolemia vs. controls in the majority of larger and well controlled studies that were matched for apo(a) isoform size. These and other observations suggested that not only LDL but also Lp(a) may be catabolized by the *LDLR* pathway. A large sib-pair study [44] and a study of South African families with familial hypercholesterolemia [45] included molecularly defined homozygous and heterozygous patients, in which KIV-2 repeat genotypes and apo(a) isoforms were determined by Westernblots. They observed a clear dose effect of defective *LDLR* alleles on Lp(a) levels: when they binned apo(a) alleles by size they found in each isoform group Lp(a) levels to increase with the number of LDL receptor mutations demonstrating a positive gene dosage effect.

The mechanism behind the elevated Lp(a) in familial hypercholesterolemia is not clear. In vitro cell culture studies and in vivo turnover studies [46] excluded the LDLR pathway as a main route of Lp(a) catabolism. This is also in line with the observation that statins that result in an overexpression of LDL receptors do not lower Lp(a) concentrations, despite a pronounced effect on LDL-cholesterol (LDL-C) concentrations [47–49]. Therefore, it is actually not clear which gene really influences Lp(a) concentrations in the case of familial hypercholesterolemia.

## Why did Genetic Studies Become the Lifeline for Lp(a) as a Risk Factor for Cardiovascular Disease?

### Association of Lp(a) Concentrations with CVD

**Fig. 3:**
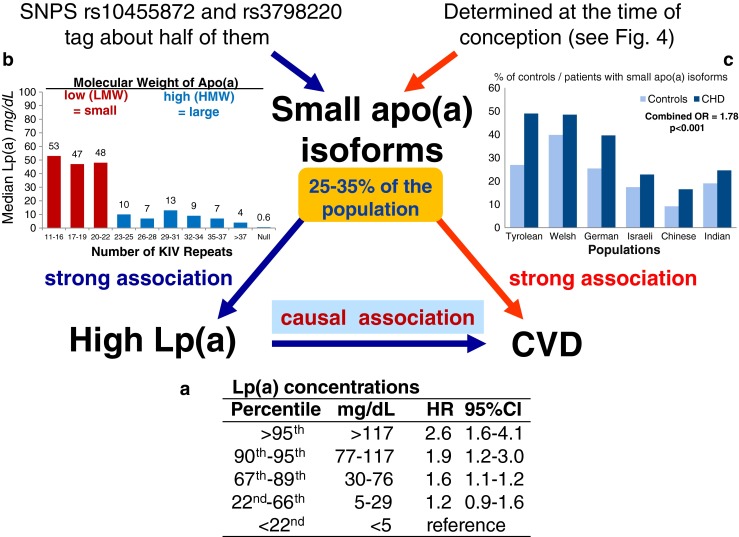
Mendelian Randomization approach to demonstrate a causal association between Lp(a) concentrations and coronary heart disease (CHD). Panel a shows the association between elevated Lp(a) concentrations and cardiovascular disease (CVD) as shown in the Copenhagen City Heart Study [53]. Panel b shows the association between the number of K-IV repeats in the *LPA* gene and Lp(a) concentrations: individuals with small apo(a) isoform have markedly higher median Lp(a) concentrations than individuals with large apo(a) isoforms. Data are derived from [107]. Panel c shows the preponderance of small apo(a) isoforms in patients with CVD when compared to controls. Data are taken from a case–control study in multiple populations [56]. Since a low number of KIV copies (11–22 copies) is associated with high Lp(a) levels and high Lp(a) levels are associated with CHD, it follows that a low number of KIV copies has to be associated with CVD if the association of Lp(a) with CVD is causal

There was a major discussion in the mid-1990s whether Lp(a) is indeed a risk factor for CVD. In the meantime there is very strong evidence that increasing Lp(a) concentrations are associated with an increasing risk for CVD. Numerous studies have been published over the last three decades. Not all were positive and this resulted in major speculations on the reasons why some were negative. These speculations included the selection of appropriate controls, selection of patients, non-standardization of assays [50, 51] and sample storage effects [52], to mention only a few. One of the largest studies up to now, the Copenhagen City Heart Study, observed a 1.6-fold increased risk for an incident myocardial infarction for concentrations between 30 and 76 mg/dL (67th – 90th percentile) compared to individuals with Lp(a) concentrations below 5 mg/dL (<22nd percentile). This risk increased to 1.90 for individuals with Lp(a) concentrations between 77 and 117 mg/dL (90th - 95th percentile) and to 2.60 for individuals with Lp(a) concentrations above 117 mg/dL (>95th percentile) [53] (Fig. 3, panel a).

### Mendelian Randomization Studies Using the KIV Repeat Polymorphism as a New Starting Point

**Fig. 4:**
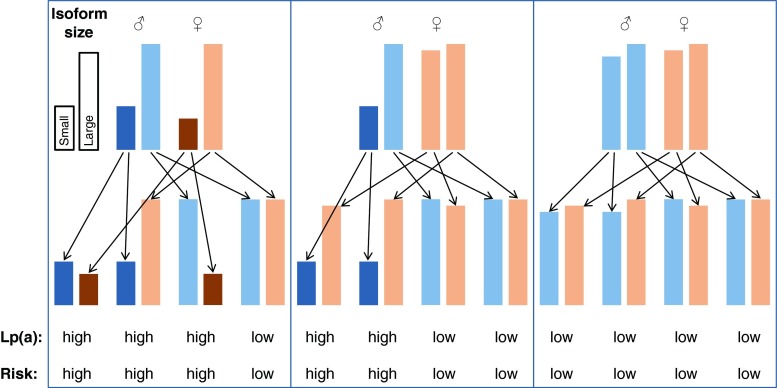
Simplified illustration how Mendelian segregation of small and large apo(a) isoforms and thereby the transmission of high or low Lp(a) concentrations and risk for CVD, respectively, works. It is randomly determined at the time of conception which of the two alleles from the father and which of the two alleles from the mother are transmitted to the offspring. The height of the bars represents the size of the isoforms. The three boxes illustrate the most common situations where both parents carry at least one small apo(a) isoform each (left box), where at least on parent carries one small apo(a) isoform (middle box) or where both parents carry only large apo(a) isoforms (right box). Underneath the typically observed Lp(a) concentrations (high or low) and the associated risk for CVD (high or low) are given. It should be pointed out that exceptions from the rules can occur since also other variants than the KIV repeat polymorphism have an influence on Lp(a) concentrations

This pronounced association, however, is at first glance not proof that Lp(a) is causally related to CVD even if the results come from several prospective studies. As already mentioned, a major discussion dominated the field in the 1990s since reverse causation could not be convincingly excluded. Reverse causation means that elevated Lp(a) in patients with CVD might be the consequence rather than the cause of the disease. Only genetic studies following the principle of the Mendelian randomization approach finally excluded reverse causation as a reason for elevated Lp(a) in CVD. This approach became quite popular during the last two decades and was applied for the first time ever with Lp(a). It is based on a triangle of observations as illustrated in Fig. 3. The first leg starts with the finding that a biomarker (in our case high Lp(a) concentrations) is associated with CVD (Fig. 3, panel a). This association could be either causal or the result of reverse causation in the sense that the outcome CVD is changing the biomarker secondarily. Furthermore, the association can be influenced by several confounders. The second leg of the triangle is that certain alleles of genes (in our case this was the number of KIV repeats determining apo(a) isoform size) have an influence on Lp(a) concentrations (Fig. 3, panel b). Figure 4 is a simplified illustration how this random assortment of alleles from parents to offsprings works. If one of the parents carries one small apo(a) isoform (usually associated with high Lp(a) concentrations) and one large apo(a) isoform (associated with low concentrations), it is randomly determined at the time of conception which of the two alleles will be transmitted to the offspring. This holds also true for the alleles of the other parents. It will thereby be determined in this early stage whether someone is exposed all their life to lower or higher Lp(a) concentrations. If Lp(a) concentrations indeed influence the risk for CVD, the triangle will be finalized by the third leg (Fig. 3, panel c) as one would expect to see a preponderance of small apo(a) isoforms in persons who develop CVD. And this was indeed the case when the first studies were published in patients with familial hypercholesterolemia with and without CHD [54] and Chinese CHD patients and controls [55] and by a multicenter-multiethnic study including >1000 CHD cases [56]. In the latter study the OR in the pooled sample was 1.78 for small apo(a) isoforms. These were actually the first studies applying the Mendelian randomization approach in practice, although they did not use this term which was only introduced in 2003 [57]. Several follow-up studies, including one in which KIV-2 repeats were determined by pulsed-field gel electrophoresis [58] and a meta-analysis of these studies confirmed the association [59]. The meta-analysis included 40 studies with 11396 cases and 46938 controls and 30 studies with 7382 cases and 8514 controls that applied broadly comparable phenotyping and analytic methods. Smaller apo(a) isoforms were associated with a twofold increased risk for CHD compared to large isoforms (RR = 2.08, 95%CI 1.76-2.58) [59]. Similar relative risks were observed for ischemic stroke (RR = 2.14; 95 % CI: 1.85-2.97) [59]. These pronounced effect sizes are probably the strongest which will ever be identified for common variants and CVD, keeping in mind that approximately 25–35 % of the population carries small apo(a) isoforms (Fig. 3). This high prevalence of small apo(a) isoforms points to a high public health relevance.

**Fig. 5:**
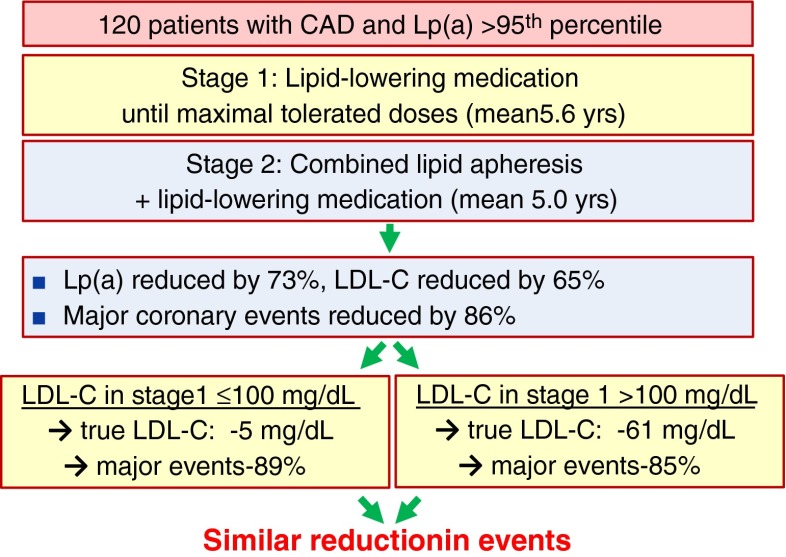
Study design and summary of the results of the study by Jaeger et al. [89] that investigated the effect of Lp(a)-lowering by lipid apheresis in very high-risk patients. “True LDL-C” is considered to be LDL-cholesterol without the cholesterol from the Lp(a) particles. The Lp(a)-derived cholesterol is not accessible for therapeutic interventions with statins and is about 45 % of the Lp(a) concentration. For extended explanation, see text. Figure taken with permission from reference [1]

### Mendelian Randomization Studies Using SNPs

The finding of the pronounced association between the number of KIV repeats and CVD is supported by further genetic studies using SNPs within the *LPA* gene region. A study of cases with myocardial infarction and controls investigated several SNPs in that gene region. Besides several other SNPs, rs10455872 and rs3798220 showed the strongest associations with myocardial infarction [37]. rs10455872 is an intronic SNP and rs3798220 results in an amino acid substitution in the protease domain of *LPA*. Both SNPs were described to be associated with small apo(a) isoforms and high Lp(a) levels. rs10455872 was associated with a 1.47-fold and rs3798220 with a 1.68-fold increased risk for CHD over non-carriers. Individuals who carried at least one risk allele of the two SNPs had a 1.51-fold elevated risk for myocardial infarction and subjects carrying two and more risk alleles of these two SNPs had a 2.57-fold increased risk [37]. These findings have been confirmed by a recent meta-analysis [60] and clearly underscore the *LPA* gene as a risk gene for CVD. This becomes even more accented in comparison to other genes for CAD detected by GWAS that were associated with odds ratios between 1.06 and 1.29 [61].

### The Disadvantage of Well-Known SNPs

These impressive findings were a turning point which brought Lp(a) back to the stage of interest. Commercial laboratories started to offer the genotyping of the two SNPs rs10455872 and rs3798220 for diagnostic purpose which is easily possible in a high-throughput manner and with a simple interpretation. They were claimed to tag carriers of small apo(a) isoforms. However, the minor allele frequencies of these two SNPs are only 7 and 2 %, respectively. It was therefore quite interesting to see the data from almost 3000 individuals of a general Caucasian population that was genotyped for these two SNPs and phenotyped for the apo(a) isoforms [62]. The results clearly showed that 47 % of all subjects expressing a small apo(a) isoform were not tagged by one of these two high-risk SNPs. Furthermore, about 11 % of all subjects carrying at least one minor allele of these two SNPs did actually not have a small apo(a) isoform. That means that these two SNPs are far from being a good surrogate for risk evaluation instead of measurement of small apo(a) isoforms since roughly half of the small apo(a) isoforms will remain undetected when only these two SNPs are genotyped. As stated figuratively, from a population perspective we might see the tip of the iceberg but we might underestimate the size [62].

In addition, the situation might be different for various ethnicities. For example, a study investigating rs3798220 and K-IV repeat number in several ethnicities revealed that this variant was not found in Africans. Allele frequencies in East and Southeast Asians ranged from 2.9 to 11.6 %, and were very low (0.15 %) in CAD cases and controls from India. The variant was neither associated with small KIV CNV alleles nor elevated Lp(a) concentrations in Asians. The study concluded that it is unlikely that this SNP confers atherogenic potential on its own and that this SNP does not explain Lp(a)-attributed risk for CAD in Asian Indians [63]. This might also be the reason why a recent GWAS for CAD Japanese patients did not find the *LPA* locus to be associated with CAD [64] although case–control studies consistently found an association of the KIV repeat polymorphism with CAD in e.g., Chinese CAD patients and controls [55, 65].

### Haplotypes Instead of Single SNPs

To increase the number of SNPs by creating haplotypes instead of single SNPs for risk prediction was a further idea which was followed by a GWAS for myocardial infarction [66]. The authors used haplotypes built from four SNPs, two in *LPA* (rs7767084 and rs10755578) and two in the neighboring genes *LPAL2* (rs3127599) and *SLC22A3* (rs2048327) gene. A rare haplotype with a frequency of roughly 2 % was associated with an 82 % higher risk (OR 1.82, 95%CI 1.57-2.12) and a more common haplotype with a frequency of about 16 % had a 20 % higher risk (OR 1.20, 95%CI 1.13-1.28) for myocardial infarction [66]. Once again, this only supports the *LPA* gene as a risk gene for CVD but does not improve risk prediction when compared to apo(a) isoforms since either only for a small group of 2 % a tremendously increased risk or for a larger group of 16 % a slightly increased risk is predicted.

### qPCR to Quantify the Sum of KIV Repeats of Both apo(a) Alleles

A further impressive genetic approach to support Lp(a) as a risk factor for CVD came from Kamstrup and colleagues who used qPCR to quantify the copy number of KIV type-2 repeats in >40,000 subjects. This method sums up the number of both apo(a) alleles and cannot distinguish each of the two alleles. They observed in the Copenhagen City Heart Study that individuals in the lower quartile of the sum of copy number in their genome had an adjusted hazard ratio for myocardial infarction of 1.50 compared to those in the highest quartile of copy number. The KIV type-2 CNV explained roughly 25 % of the variability in Lp(a) levels [53], which is lower than in other studies of European populations using methods such as apo(a) isoforms by Westernblot or separated alleles by pulsed-field gel electrophoresis. However, these estimates in risk and unexplained variability determined by qPCR are most likely an underestimation due to the special characteristics of qPCR (see below).

All these approaches and data together provide pronounced genetic evidence that Lp(a) is an emerging genetic risk factor for cardiovascular disease that is independent of other classical risk factors including lipids.

## Advantages and Disadvantages of Various Methods for Risk Prediction

The measurement of Lp(a) concentrations comes probably most closely to the component which actively contributes to risk. It measures the protein of interest and can be done in high-throughput. Since a protein and not a genetic polymorphism is measured, no genetic counseling is required in most countries. A disadvantage is the fact that the Lp(a) measurement is still insufficiently standardized [50, 51, 67, 68]. This is caused by the repetitive structure of the KIV copies and most assays have no proof that the applied antibodies are directed against unique non-repetitive elements of apo(a). Although Lp(a) seems to be relatively stable throughout life [4, 69], there are several conditions known that influence Lp(a) concentrations secondarily (for review see [1]).

Westernblot analysis of the number of KIV repeats is quite laborious and only few laboratories have established the method of SDS agarose gel electrophoresis. Nevertheless, from all methods that investigate apo(a) genetic polymorphisms, it provides the most comprehensive information and is very suitable for risk prediction. This has to be seen in the context of a peculiarity of this risk factor: roughly 30–50 % of all individuals show only one isoform in their plasma although 95 % of the population should be heterozygous and show two isoforms. Part of this discrepancy can be explained by the resolution of the method which does not allow separating two isoforms that have only a difference of 1–2 KIV repeats. However, the major part of this discrepancy is explained by the fact that many subjects express only one apo(a) isoform as protein although two isoforms are expressed at the DNA level [26, 27]. Factors which influence the protein expression or non-expression are currently insufficiently understood. The Westernblot analysis of KIV repeats is among all polymorphism methods the most informative one, since it considers only the variants that make it to the plasma level. The other non-expressed isoform, although scientifically interesting, might not contribute to the risk. A further advantage of this method is that genetic counseling is not required in most of the countries since not DNA but the protein is investigated. Furthermore, it is more informative than Lp(a) plasma concentrations for risk prediction in certain patient groups where Lp(a) concentration changes secondarily in a substantial manner as this is the case in patients with chronic kidney disease [1, 2, 70–73].

Pulsed field gel electrophoresis provides the number of KIV repeats at the DNA level for each of the two apo(a) isoforms and can be used for risk prediction although it might be less informative than Westernblot analysis. This can be explained by the fact, that the expression level of each protein isoform is not investigated by this method. The method is very laborious and takes up to 2 weeks. Most importantly, it requires a special, time and costly DNA preparation since non-fragmented DNA is required. This is no longer the case when DNA is already extracted from full-blood with conventional DNA extraction methods since they generate DNA fragments of mostly 30 to 100 kb in size. Therefore, the analysis of ancient DNA samples is not possible. The method ignores the expression status of the protein and is partially reliable for risk prediction. Furthermore, it requires genetic counseling in most countries.

The qPCR (see above) provides the sum of the KIV repeats of the two alleles and some information gets lost since the expression status of the protein is not considered. The consequence is that individuals with one very short KIV-2 repeat allele and one very large allele end up in the same category as individuals with two intermediate copy number alleles. These two situations are, however, associated with very distinct Lp(a) concentrations. On the other hand, qPCR is a high-throughput method suitable for large epidemiological studies but requires genetic counseling if analysis is done for single individuals.

SNPs such as rs10455872 and rs3798220 became quite popular recently since they are easy to analyze in the routine lab. These two SNPs tag only half of the small apo(a) isoforms resulting in a large frequency of false negatives and are therefore not as good for risk prediction. They ignore the expression status of the protein and genetic counseling might be required.

## One Grey Spot on Lp(a) - Is a Lowering of Lp(a) Beneficial?

After the strong genetic support that Lp(a) is a risk factor and not only a risk marker for CVD, a burning question is whether therapeutic lowering of Lp(a) indeed also lowers the CVD risk. Despite therapeutic options being on the horizon, it turns out that this key question is not easy to investigate since there are no simple means to lower Lp(a) effectively without changing other lipid risk factors at the same time. It will be difficult, if not impossible to disentangle effects of LDL-C and Lp(a) lowering as well as other changes in lipoproteins. Several of the options fell away in the meanwhile since the further development of some drugs was stopped for other reasons not necessarily connected to Lp(a).

Niacin was considered by many researchers as kind of magic bullet since it increases HDL-cholesterol markedly and as a further positive effect it lowers triglycerides, LDL-C and also Lp(a). Recent turnover studies demonstrated that niacin decreases production rates of apo(a) [74, 75]. However, two clinical trials in patients with optimally low levels of LDL-C failed to show any further clinical benefit on CVD events when niacin was added to simvastatin [76, 77]. We do not really know whether niacin would be beneficial in patients with isolated Lp(a) elevations since we are missing trials that were designed to elucidate this question. Furthermore, niacin is no longer available in most countries after the two studies have failed [76, 77].

Cholesteryl ester transfer protein (CETP) inhibitors are a further drug class that was also not designed to lower Lp(a). CETP is a major player in the reverse cholesterol transport and inhibitors of that protein markedly increase HDL-cholesterol concentrations. They also lower Lp(a) by 20–40 % [78]. Five drugs have been developed and they increased HDL-cholesterol by up to more than 100 %. However, phase-III trials resulted in premature stops of three of the trials either due to side-effects (torcetrapib) or futility (dalcetrapib and evacetrapib).

Other treatment options target the apo(a) synthesis using antisense oligonucleotide against apolipoprotein B (mipomersen) [79, 80] or apo(a) [81], thyroid hormone analogue therapies [82] or microsomal triglyceride transfer protein inhibitors [83], but clinical outcome data are still missing. Recently, data from a randomized, double-blind, placebo-controlled, phase 1 study of a second-generation antisense drug designed to reduce the synthesis of apolipoprotein(a) in the liver have been published. This therapy resulted in dose-dependent, mean percentage decreases in plasma Lp(a) concentration between 40 and 78 % [81]. If this therapy comes to phase 3 trials, it will be one of the few possibilities that might be able to investigate whether an isolated lowering of Lp(a) indeed lowers CVD outcomes as well.

A major hope for lowering Lp(a) concentrations are human monoclonal antibodies to proprotein convertase subtilisin/kexin type 9 (PCSK9) [84]. PCSK9 is a protein synthesized and secreted by hepatocytes that binds to the LDL-receptor to mark it for lysosomal degradation. Inhibitors of PCSK9 not only substantially lower LDL-C but also Lp(a) concentrations. This finding was a major surprise since statins are also known to have a pronounced effect on LDL-C by upregulation of the LDL receptor but not on Lp(a) concentrations [47–49]. From statin trials it has been concluded that Lp(a) does not bind to the LDL receptor. However, contrary to expectations, inhibitors of PCSK9 also result in a higher availability of LDL receptors but show a significant reduction of Lp(a) in a dose-dependent manner in subjects with hypercholesterolemia who are already on lipid-lowering therapy [85]. The reduction in Lp(a) is proportional to the baseline Lp(a) [86]. The precise mechanism via which anti-PCSK9 lowers Lp(a) remains to be elucidated. It could well be that PCSK9 also regulates a receptor other than the LDL receptor that is responsible for the uptake of Lp(a). Although the main outcome trials are still under way, recent post-hoc and pre-specified exploratory data analyses of outcome studies revealed that the use of PCSK9 inhibitors in addition to standard therapy and/or statin therapy at the maximum tolerated dose significantly reduced LDL-C levels and reduced the incidence of cardiovascular events [87, 88]. It has to be seen whether trials in patients with isolated Lp(a) elevations will be performed in the future and whether they will show an effect on CVD outcomes.

The best evidence for a beneficial effect of Lp(a)-lowering that we currently have, comes from apheresis studies. Again, these studies are at first glance not perfect since this procedure removes Lp(a) and LDL-C simultaneously. However, a study by Jaeger and colleagues [89] used a sophisticated design (Fig. 5) and selected patients with Lp(a) levels above the 95th percentile who continued to experience a high rate of major adverse coronary events (MACE) despite receiving the maximum tolerated doses of lipid-lowering drug treatment and successful lowering of LDL-C in the first and retrospective phase of the study that lasted on average 5.6 years. In the second and prospective phase, patients underwent LDL apheresis besides the lipid-lowering drug treatment for on average 5 years. In this phase Lp(a) was lowered on average by 73 % and the rate of MACE decreased dramatically by 86 % compared to the first study phase. To exclude that this decrease in MACE was only due to further lowering of LDL-C levels, the authors analyzed a subgroup of patients who had already LDL-C ≤100 mg/dL before the start of the apheresis phase. This “measured LDL-C” in their plasma was mainly cholesterol due to their very high Lp(a) concentrations and the “true LDL-C” was on average only 23 mg/dL. Consequently, apheresis selectively and dramatically lowered Lp(a) in these patients and the true LDL-C dropped only from 23 to 18 mg/dL. Most importantly, the effect on MACE in this subgroup was of the same magnitude as in the subgroup that started with measured LDL-C concentrations above 100 mg/dL (−89 vs. -85 %, respectively). Therefore, the very small reduction in the true LDL-C in the low LDL-C group by 5 mg/dL is too small to explain this dramatic reduction in MACE by 89 % [89]. In a further study in patients with isolated elevation of Lp(a), progressive cardiovascular disease, and maximally tolerated lipid-lowering medication, lipid apheresis effectively lowered the incidence rate of cardiovascular events [90].

Further promising evidence that an isolated lowering of Lp(a) is beneficial comes from a small study in patients with CHD, Lp(a) >50 mg/dL, and LDL-C below 100 mg/dL and on chronic statin therapy. Half of the patients underwent a specific Lp(a) apheresis on a weekly basis. Both patient groups received a quantitative coronary angiography analysis of percent diameter stenosis before and 18 months after start of therapy. Lp(a) decreased on average by 73 % without significant changes in true LDL-C. The mean percent diameter stenosis at 18 months as primary efficacy end-point decreased by 5 % in the Lp(a) intervention group and increased by 5 % in the control group that received only statins [91].

## Three Major Surprises Concerning Lp(a) During Recent Years

### Lp(a) and Aortic Valve Calcification, Aortic Valve Stenosis and Heart Failure

A recent GWAS identified rs10455872 of the *LPA* gene to be significantly associated with the presence of aortic-valve calcification with an odds ratio per allele of 2.05 (95%CI 1.63–2.57). In prospective analyses, the *LPA* genotype was associated with incident aortic stenosis and aortic-valve replacement [92]. In large studies from the general population, elevated Lp(a) levels and corresponding *LPA* risk genotypes predicted increased risk of incident aortic valve stenosis and the risk estimates were very similar to the observations for myocardial infarction [93, 94]. Furthermore, Lp(a) levels were also associated with aortic valve calcification in asymptomatic patients with familial hypercholesterolemia who were free of symptomatic CVD or symptoms suggestive of ischemic heart disease at the time of recruitment [95]. A prospective study in 220 patients with mild to moderate aortic stenosis described that progression of aortic stenosis was faster in the top tertiles of Lp(a) concentrations as well as oxidized phospholipids [96].

Lp(a) transports oxidized phospholipids with a high content in lysophosphatidylcholine. It has recently been demonstrated that autotaxin transforms lysophosphatidylcholine into lysophosphatidic acid that promotes deposition of hydroxyapatite of calcium in aortic valve. Autotaxin is transported in the aortic valve by Lp(a) and is also secreted by valve interstitial cells. This promotes inflammation and mineralization of the aortic valve [97].

The most recent findings published were on an association between Lp(a) concentrations and heart failure in more than 98000 individuals from the general population of Denmark [98]. In 4122 of them heart failure had been diagnosed during the observation period. Lp(a) concentrations in the upper tertile were significantly associated with heart failure compared to the lowest tertile and this association was graded within the upper tertile: HR = 1.24 (95%CI: 1.08-1.42) for the 67th to 90th percentiles, HR = 1.57 (95%CI: 1.32-1.87) for the 91st to 99th percentiles, and HR = 1.79 (95%CI: 1.18 to 2.73) for levels >99th percentile compared to the lowest tertile corresponding to a population-attributable risk of 9 %. *LPA* risk genotypes that are associated with high Lp(a) concentrations were in line with this association. When participants with a myocardial infarction or aortic valve stenosis were excluded, the risk estimates were attenuated and mediation analysis revealed that 63 % of heart failure risk was mediated via myocardial infarction or aortic valve stenosis [98].

### Lp(a) and Venous Thromboembolism

The high homology of apo(a) and plasminogen suggested a link to the fibrinolytic system with blood clotting and fibrinolysis. It was therefore not clear whether the culprit is the development of atherosclerosis or thrombosis. Again a Mendelian randomization study in more than 41000 individuals has shed some light into the dark. Neither Lp(a) tertiles nor the sum of K-IV repeats were associated with the risk of venous thrombosis in general population studies [99]. A further study including 4607 cases with venous thromboembolism investigated the two *LPA* variants rs10455872 and rs3798220 and did also not find an association [100]. However, both studies found associations with CVD [99, 100].

However, a meta-analysis of eight studies including 589 children with venous thromboembolism and 1441 controls described elevated Lp(a) levels to be associated with an odds ratio of 4.50 (3.19–6.35) for first onset venous thromboembolism. The association with 135 recurrent cases of venous thromboembolisms was not statistically significant [101].

### Lp(a) and Diabetes Mellitus

When Mora et al. described an association between very low Lp(a) concentrations and type 2 diabetes mellitus (T2DM) a few years ago, it was a major surprise [102]. The highest risk was found for Lp(a) <1 mg/dL (lowest 2.6 % of observations) with an odds ratio of 1.57 when compared to higher values. When the risk group was changed to Lp(a) concentrations below 4 mg/dL (equals the lowest quintile), the size of the risk was markedly attenuated to 1.18 [102]. These findings were contrary to expectations and the study could not clarify the causality between low Lp(a) levels and T2DM and could not exclude “reverse causation”. Kamstrup and Nordestgaard could not only replicate the association between low Lp(a) concentrations and T2DM in a large Danish study but also demonstrated that the highest quintile of the sum of the KIV-2 repeats from the two apo(a) alleles is associated with T2DM [103]. This quintile combines large isoforms and is associated with low and medium Lp(a) concentrations and therefore supports causality and excludes that the association between low Lp(a) concentrations and T2DM is merely due to reverse causation or confounding. Two other studies followed and confirmed the association between low Lp(a) concentrations and T2DM [104, 105]. An investigation in more than 10000 Chinese individuals extended the findings and found an association not only between low Lp(a) concentrations and prevalence of T2DM but also prevalent prediabetes, insulin resistance and hyperinsulinemia [105].

Mendelian randomization studies that investigated an association between the SNP rs10455872 and T2DM caused more confusion than support for causality [103, 104]. The carrier-status of this SNP is already well known for its association with CVD since it is associated with high Lp(a) concentrations [37]. The authors of these two studies [103, 104] used the non-carrier status of rs10455872 as a surrogate for genetically determined low Lp(a) concentrations to investigate the causality for the association between low Lp(a) concentrations and T2DM. Since they did not find an association between the non-carrier status of rs10455872 and T2DM, a causal role of Lp(a) concentrations has been called into question [103, 104]. As we discussed recently [106], the non-carrier status of rs10455872 includes already 86 % of the population with a very wide range in Lp(a) concentrations and with the inclusion of large and small apo(a) isoforms [103]. We have to bear in mind that the association of Lp(a) and T2DM is especially seen in subjects with ultra-low Lp(a) concentrations (e.g., <1 mg/dL) and was already weaker, when the low class was extended to the lowest quintile [103]. With other words, the non-carrier status of the SNP explains the wrong range of the Lp(a) values which might “dilute” the association of very low Lp(a) levels with T2DM. Therefore, rs10455872 is obviously an imprecise instrument to support or exclude causality of low Lp(a) levels for T2DM [106].

One might ask the question whether it would be counterproductive to lower Lp(a) to avoid CVD events but simultaneously increase the risk for T2DM? This is probably not the case since the risk for T2DM is especially elevated for persons with very low Lp(a) concentrations and an Lp(a)-lowering therapy will not bring Lp(a) in these concentration ranges.

## Conclusions

Lp(a) plays a major role for complex diseases such as cardiovascular disease and type-2 diabetes mellitus. Genetic studies helped to elucidate the causal role of this lipoprotein for these diseases. Especially this knowledge makes it reasonable to search for therapeutic options to lower Lp(a) concentrations to decrease the risk for cardiovascular disease.
